# Development and validation of an HPLC method for determination of rofecoxib in bovine serum albumin microspheres

**DOI:** 10.3906/kim-1912-45

**Published:** 2020-06-01

**Authors:** Esra DEMİRTÜRK, Emirhan NEMUTLU, Selma ŞAHİN, Levent ÖNER

**Affiliations:** 1 Department of Pharmaceutical Technology, Faculty of Pharmacy, Çukurova University Turkey; 2 Department of Pharmaceutical Technology, Faculty of Pharmacy, Hacettepe University Turkey; 3 Department of Analytical Chemistry, Faculty of Pharmacy, Hacettepe University Turkey

**Keywords:** Rofecoxib, microspheres, HPLC, validation

## Abstract

A simple and reliable HPLC method was developed and validated for determination of rofecoxib in bovine serum albumin microsphere. The analyses were performed on a C18 column (150 x 4.6 mm, 5 μm particle size) at room temperature with UV detection at 272 nm. The mobile phase was composed of acetonitrile-0.1% o-phosphoric acid solution in water (1:1, v/v) mixture, and flow rate was set to 1 mL/min. The method was validated according to the international guidelines with respect to stability, linearity range, limit of quantitation and detection, precision, accuracy, specificity, and robustness. The detection and quantification limit of the method were 1.0 μg/mL and 2.5 μg/mL, respectively. The method was linear in the range of 2.5–25 μg/mL with excellent determination coefficients (R2 >0.99). Intra-day and inter-day precision (<1.76% RSD) and accuracy (<0.55 % Bias) values of the method also fulfilled the required limits. It was concluded that the developed method was accurate, sensitive, precise, and reproducible according to the evaluation of the validation parameters. The applicability of the method was confirmed for in vitro quantification of rofecoxib in bovine serum albumin microspheres.

## 1. Introduction

**Figure 1:**
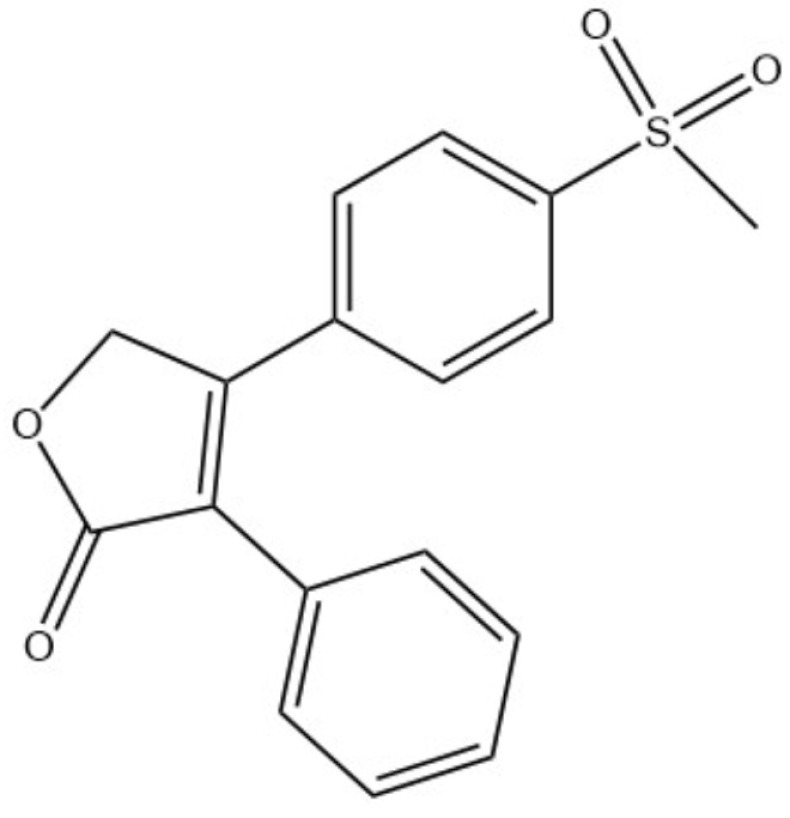
Chemical structure of rofecoxib.

Rofecoxib is a nonsteroidal antiinflammatory drug (NSAID) that shows, antiinflammatory, analgesic, and antipyretic effects (Figure 1). These drugs therapeutically act via the inhibition of the enzyme cyclooxygenase (COX). Rofecoxib which is a selective cyclooxygenase-2 inhibitor (COX-2), is used for osteoarthritis symptoms, dysmenorrhea, and acute pain. Because of increased risk of coronary thrombosis and cerebrovascular risk after its chronic use (about 18 months) it was voluntarily withdrawn from the global markets. However, rofecoxib is currently used for research purposes comprising characterization studies, preparation of new formulations, and also in clinical studies. Rofecoxib is a Class II compound according to Biopharmaceutics Classification System (low solubility and high permeability) and has a long half-life (t_1/2_ = 17 h). Therefore, it is used as a model drug in the formulation studies of controlled release dosage forms, and also in new drug delivery systems [1–3].

The preclinical studies on NSAID nanoformulations have been shown to reduce the toxicity while enhancing the bioavailability of incorporated NSAIDs at equal doses as compared to conventional NSAID formulations. Furthermore, compared to conventional formulations, a number of nanoformulations were able to sustain the release of the loaded NSAIDs, and improve the pharmacodynamics of the encapsulated drug in preclinical models of inflammatory diseases. These advantages have been demonstrated using various routes of administration including oral, parenteral, ocular, transdermal, and others for the nanoformulations. We previously prepared bovine serum albumin (BSA) microspheres containing rofecoxib for oral administration by emulsion polymerization technique to minimize the side effects and to extend the release time. A biodegradable and nontoxic polymer BSA was chosen for the preparation of microspheres. An aqueous solution of glutaraldehyde (25% w/v, 4 mL) was used as the crosslinking agent with 30 min of crosslinking time. Results revealed that microparticular dosage forms with well-controlled release were obtained with better-sustained release up to a period of 18 h. The details of the preparation and characterization of BSA microspheres were given in our previous study [4].

In the literature, various methods were reported for the analysis of rofecoxib in bulk drug and pharmaceutical dosage forms [5,6] including High-Performance Liquid Chromatography (HPLC), square wave voltammetry [7], capillary electrophoretic [8], and spectrophotometric [9] methods. Of these methods, HPLC method is the most commonly used for the determination of rofecoxib in plasma [10–14]. Survey of the literature showed that there is no method available for the analysis of rofecoxib in the presence of BSA. Therefore, this study was designed to develop a simple, fast, and validated HPLC method for determination of rofecoxib in BSA microspheres. Since there are many types of HPLC methods depending on detector and column types, it is a challenge for the researchers to choose a suitable method for their drug delivery systems [15,16]. In this study, the optimum chromatographic and analytical parameters were investigated and the method validation studies were performed according to FDA guideline in line with the bioanalytical method validation procedure. The method was validated as to linearity, accuracy, precision, specificity, sensitivity, and stability parameters [17–21].

## 2. Materials and methods

### 2.1. Materials

Rofecoxib was obtained from Dr. Reddy’s (India), BSA, acetonitrile, and o-phosphoric acid were purchased from Sigma (USA) and Merck (Germany). Deionized water was obtained from the Milli-Q water system (Barnstead, USA), and used for preparation of all standard solutions and buffers. All other chemicals used were of analytical grade.

### 2.2. Methods

#### 2.2.1. Instrumentation

An HP Agilent 1100 series HPLC system (USA) equipped with solvent pump, injection valve and a diode-array detector was used. The separation of compound was achieved with a C18 column (150 x 4.6 mm, 5 μm particle size). The mobile phase consisted of acetonitrile-0.1% o-phosphoric acid solution in water (1:1, v/v) mixture, and delivered at a flow rate of 1mL/min which gave the best resolution within acceptable analysis time and column back pressure. The injection volume was 10 μL. The UV detector was operated at 272 nm.

#### 2.2.2. Standard solutions

Both poor water solubility and wettability of COX-2 inhibitors cause difficulties in formulation development phase and subsequently results in a difference in oral bioavailability. Since there is no ionizable group in the rofecoxib structure, it is not ionized at any pH value and no effect of pH is observed on its solubility. Therefore, studies are planned to increase and evaluate the solubility of rofecoxib with different solvents and solventcosolvent mixtures. The stock solution of rofecoxib (25 μg/mL) was prepared in deionized water containing 2% sodium dodecyl sulphate (SDS). The stock solution was then diluted with the same medium to obtain standard solutions within the concentration range of 2.5–25 μg/mL.

#### 2.2.3. Method validation

The developed HPLC method was validated as to linearity, accuracy, precision (intra-assay precision and reproducibility), specificity, sensitivity and stability [22].

#### 2.2.4. Linearity

The linearity of the method was determined by spiking 10 different concentrations within the concentration range of 2.5–25 μg/mL. Six calibration curves were carried out. The calibration equation is characterized by determination coefficient, slope, and intercept.

#### 2.2.5. Precision and accuracy

The intra- and inter-day precision studies were carried out for assessment of the assay precision. Three different concentrations (2.5, 12, and 25 μg/mL) within calibration range were analysed 6 consecutive days (inter-day) and 6 times within the same day (intra-day) to determine the precision of the method. The inter- and intra-day accuracies of the method were also determined at the same 3 concentrations.

#### 2.2.6. Specificity

The specificity of the analytical method was assessed by injecting drug sample into the HPLC system. For this purpose, the solutions of the excipients used in the microsphere formulations were prepared in the same concentrations and chromatograms were taken in order to examine whether they gave peaks under the same conditions as the active substance.

#### 2.2.7. Sensitivity

Lower limit of detection (LOD), the lowest amount of analyte in a sample which can be detected, and lower limit of quantitation (LLOQ), the lowest amount of analyte in a sample which can be quantitatively determined with suitable precision and accuracy, were determined to evaluate the sensitivity of the analytical method.

#### 2.2.8. Stability

The photo stability of the samples at 2 different concentrations (2.5 and 25 μg/mL) were evaluated by exposing the samples to daylight or not. The results were compared with initial concentrations.

#### 2.2.9. Application of the developed method

The developed and validated method was used to determine the rofecoxib content of BSA microsphere formulation. Briefly, BSA microspheres containing rofecoxib were prepared by emulsion polymerization method. Laser diffraction method was used for measurement of particle size (Helos Particle Size Analysis; Sympatec, England). Before the size analysis, a small amount of RXB microspheres was dispersed in deionized water containing Tween 80 (0.1%, w/v), and sonicated in an ultrasonic bath for 1 min. Particle size analysis was carried out by 3 consecutive measurements for each sample, and results were expressed as mean ±SD. For stabilization of resulting microspheres, 1, 2, or 4 mL of glutaraldehyde aqueous solution (25%, w/v) in ether (100 mL) was added to the microspheres (coded as BSA-1, BSA-2, and BSA-4, respectively), mixed, and then centrifuged. After the washing process, the excess oil was removed and the microspheres were dried at room temperature [19]. To determine the encapsulation efficiency of microspheres, rofecoxib containing BSA microspheres were accurately weighed (10 mg), mixed with 5 mL of glacial acetic acid (1N), and stored at 4 °C for 12 h before analyses of drug content. The volume was made up to 50 mL with SDS (2%) containing distilled water, sonicated for 1 h and filtered through a 0.45 μm membrane filter. The rofecoxib amount was determined by the developed and validated HPLC method. Preparation and encapsulation efficiencies were calculated using Equations 1 and 2, respectively (19).

(1)PreparationEfficiency=(Weight of microspheres at the end of the productionTotalweight of active substance and polymer used in production)x100

(2)EncapsulationEfficiency=(Weight of the drug in microspheresTheoreticalweight of the drug)x100

## 3. Results and discussion

### 3.1. Optimization of chromatographic conditions

Method development was started with selecting the suitable wavelength to keep the baseline noise minimum, and achieve optimum system suitability parameters [14,15]. It was determined that rofecoxib has a maximum absorbance in 272 nm wavelength. Column selectivity for the separation of all related substances is critical. Rofecoxib was well retained and separated with comparatively sharp peaks using the C18 column (150 x 4.6 mm, 5 μm particle size). Several mobile phases with different compositions were examined for their efficiency in resolution, and finally acetonitrile-0.1% o-phosphoric acid solution in water (1:1, v/v) mixture was selected as it yielded the best separation. Based on the analysis time and column back pressure, the mobile phase flow rate and the temperature of the column were fixed at 1 mL/min and room temperature respectively [10,11]. The optimum conditions for the HPLC method are given in Table 1.

**Table 1:** Optimum conditions for HPLC analysis.

Wave length	272 nm
Column	C18 (150 x 4.6 mm, 5 μm particle size)
Mobile phase	Acetonitrile-0.1% o-phosphoric acid solution in water (1:1, v/v)
Flow rate	1 mL/min
Temperature	Room temperature

### 3.2. Validation of the method

#### 3.2.1. Specificity

**Figure 2:**
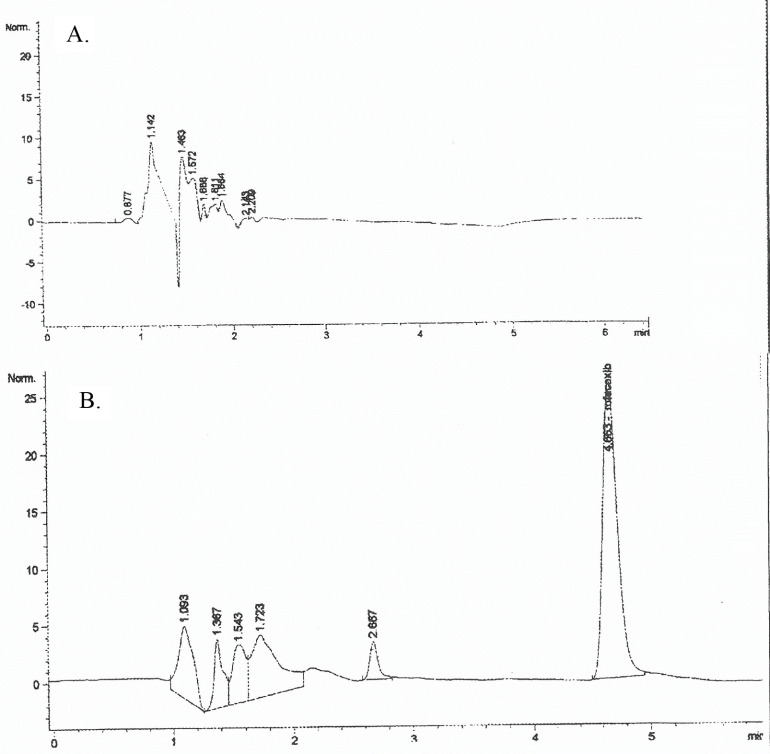
HPLC chromatogram of (A) blank formulation and (B) rofecoxib (5μg/mL).

Specificity is the ability of an analytical method to differentiate and quantify the analyte in the presence of other components in the sample. Complete resolution of rofecoxib (5μg/mL) from its related compounds with no apparent shoulders (Figure 2) confirmed the specificity of the described method [12].

#### 3.2.2. Linearity

Calibration curves for rofecoxib (n = 6) were constructed by plotting the peak area against the corresponding nominal concentrations (2.5–25 μg/mL). Linearity of the method was demonstrated by the calibration equation and determination coefficient (Table 2). The method was found to be linear within the concentration range of 2.5–25 μg/mL, and also the determination coefficient over 0.999 was taken as an indication of linearity.

**Table 2:** 

Parameter	Rofecoxib
Calibration equation*	y = 32.322x + 1.1264
Determination coefficient (R^2^)	0.999
Linearity range (μg/mL)	2.5-25
Number of points	10
LLOQ (μg/mL)	2.5
LOD (μg/mL)	1.0

* Linear regression analysis with a calibration equation of y = ax + b in which x is the concentration in μg/mL of compound and y is the peak area.

#### 3.2.3. Sensitivity

The sensitivity of the analytical method was evaluated by determining LOD; (signal to noise ratios of 3:1) and LLOQ; (signal to noise ratios of 10:1). The LOD value of 1μg/mL and LLOQ value of 2.5 μg/mL were verified the sensitivity of the analytical method (Table 2). LLOQ was taken as lowest concentration of rofecoxib that could be quantitively determined with acceptable accuracy and precision [22].

#### 3.2.4. Accuracy

The accuracy of the method was determined at 3 different concentrations (2.5, 12.5, and 25 μg/mL), and the results were expressed as the percentage of difference between the added and measured concentrations (Table 3). The Bias values which are less than 2% was taken as an indication of sufficient accuracy of the developed method [23].

**Table 3:** Accuracy results.

Added concentration (2.5, 12.5, 25 μg/mL)	Measured concentration (μg/mL)	Percentage (%)				
	2.5	12.54	25.09	100	100.32		100.36
	2.46	12.43	24.91	98.4	99.44	99.64
	2.44	12.48	25.06	97.6	99.84	100.24
	2.53	12.56	24.92	101.2	100.48	99.68
	2.52	12.58	24.94	100.8	100.64	99.76
	2.55	12.00	24.96	102	96.00	99.84
	2.5	12.432	24.98	100	99.453	99.92
	0.042	0.219	0.076	1.697	1.749	0.305
	1.697	1.758	0.305	1.697	1.758	0.305
Bias	0.00	0.55	0.08	0.00	0.55	0.08

#### 3.2.5. Precision

Relative standard deviation (RSD) values calculated for repeatability and reproducibility (Table 4) studies were less than 2% indicating that the method is working with the required precision [23–26].

**Table 4:** Repeatability and reproducibility results.

Sample number	Repeatability			Reproducibility		
	Concentration (μ g/mL)			Concentration (μ g/mL)		
	2.5	12.5	25	2.5	12.5	25
1	2.52	12.47	25.00	2.50	12.54	25.09
2	2.50	12.48	24.97	2.46	12.43	24.91
3	2.48	12.54	25.04	2.44	12.48	25.06
4	2.46	12.48	24.96	2.53	12.56	24.92
5	2.50	12.51	24.98	2.52	12.58	24.94
6	2.53	12.53	25.00	2.55	12.00	24.96
Mean	2.50	12.51	24.99	2.50	12.43	24.98
SD	0.03	0.03	0.03	0.04	0.22	0.08
RSD({\%})	1.03	0.23	0.11	1.70	1.76	0.31

#### 3.2.6. Stability

The photo stability of the samples was evaluated at 2 different concentrations (2.5 and 25 μg/mL) by exposing the samples to daylight or not. Rofecoxib concentration in the samples were determined immediately and 6 h after preparation. The RSD values showed that rofecoxib was stable during the whole analytical procedure (Table 5).

**Table 5:** 

		Protected from daylight		Not protected from daylight	
Time (h)	Added concentration (μg/mL)	Measured concentration (μg/mL)	RSD (%)	Measured concentration (μg/mL)	RSD (%)
0	2.5	2.50 ±0.01	0.715	2.50 ±0.01	1.15
6	2.5	2.48 ±0.02		2.46 ±0.03	
0	25	24.98 ±0.15	0.557	24.97 ±0.16	0.62
6	25	24.99 ±0.14		24.86 ±0.14	

### 3.3. Application of the method

The mean particle sizes for microsphere formulations prepared by different amounts of crosslinking agent were determined as 13.77 ±1.17, 13.70 ±1.24, and 13.48 ±2.11 μm, respectively for the formulations prepared by using 1, 2, and 4 mL crosslinking agent. Preparation efficiencies of the formulations were calculated as 55.65 ±1.48%, 50.91 ±1.36%, and 52.36 ±1.14%. HPLC assay showed that encapsulation efficiencies of BSA-1, BSA-2, and BSA-4 microspheres were 33.99 ±0.68%, 32.45 ±0.76%, and 35.20 ±1.32%, respectively. According to these results, the validated method can easily be used for the in vitro characterization of rofecoxib from BSA microspheres.

## 4. Conclusion

A new HPLC method was developed and validated for determination and quantification of rofecoxib in the presence of BSA. The method was successfully validated, and all results obtained confirmed selectivity, linearity, sensitivity, precision, and accuracy of the proposed method. Compare to other methods reported in the literature, the developed method was simple, fast and easily validated. Also, all validation results clearly demonstrated that reliable data can be obtained in further experiments such as formulation development and quality control studies for rofecoxib.
